# Oxytocin and Opioid Receptor Gene Polymorphisms Associated with Greeting Behavior in Dogs

**DOI:** 10.3389/fpsyg.2017.01520

**Published:** 2017-09-07

**Authors:** Enikő Kubinyi, Melinda Bence, Dora Koller, Michele Wan, Eniko Pergel, Zsolt Ronai, Maria Sasvari-Szekely, Ádám Miklósi

**Affiliations:** ^1^MTA-ELTE Comparative Ethology Research Group Budapest, Hungary; ^2^Department of Ethology, Eötvös Loránd University Budapest, Hungary; ^3^Advanced Dog Behavior Solutions, LLC Westport, CT, United States; ^4^Department of Medical Chemistry, Molecular Biology and Pathobiochemistry, Semmelweis University Budapest, Hungary

**Keywords:** dog, wolf, gene-behavior association, OXTR, OPRM1, greeting behavior

## Abstract

Meeting humans is an everyday experience for most companion dogs, and their behavior in these situations and its genetic background is of major interest. Previous research in our laboratory reported that in German shepherd dogs the lack of G allele, and in Border collies the lack of A allele, of the oxytocin receptor gene (OXTR) 19208A/G single nucleotide polymorphism (SNP) was linked to increased friendliness, which suggests that although broad traits are affected by genetic variability, the specific links between alleles and behavioral variables might be breed-specific. In the current study, we found that Siberian huskies with the A allele approached a friendly unfamiliar woman less frequently in a greeting test, which indicates that certain polymorphisms are related to human directed behavior, but that the relationship patterns between polymorphisms and behavioral phenotypes differ between populations. This finding was further supported by our next investigation. According to primate studies, endogenous opioid peptide (e.g., endorphins) receptor genes have also been implicated in social relationships. Therefore, we examined the rs21912990 of the OPRM1 gene. Firstly, we found that the allele frequencies of Siberian huskies and gray wolves were similar, but differed from that of Border collies and German shepherd dogs, which might reflect their genetic relationship. Secondly, we detected significant associations between the OPRM1 SNP and greeting behavior among German shepherd dogs and a trend in Border collies, but we could not detect an association in Siberian huskies. Although our results with OXTR and OPRM1 gene variants should be regarded as preliminary due to the relatively low sample size, they suggest that (1) OXTR and OPRM1 gene variants in dogs affect human-directed social behavior and (2) their effects differ between breeds.

## Introduction

To investigate the comparative biology of human behaviors and uncover the genetic background of behavior disorders, in the past decade several research groups have studied the effects of gene variants on dog behaviors (Hall and Wynne, 2012). Oxytocin and opioid receptor genes are among the candidates for nervous system pathways that regulate canine social behavior toward humans.

Oxytocin is an evolutionarily highly conserved neuropeptide that plays an important role in various complex social behaviors, such as social cognition, trust, attachment, and sociability (Donaldson and Young, 2008). Oxytocin receptor genes have also been investigated in other domestic species; for example, in cats researchers found that the G738A OXTR SNP was associated with the personality trait “Roughness” (irritable, dominant, forceful, and moody behavior), as reported by owner questionnaire (Arahori et al., 2016). However, oxytocin has received the most extensive interest in how it may modulate dogs' behavior toward humans (Buttner, 2016; Jensen et al., 2016; Thielke and Udell, 2017). For example, intranasal administration of oxytocin was found to enhance motivation to approach and affiliate with owners (Romero et al., 2014), and increase looking back at human partners in a situation involving threatening behavior signals by a human (Hernádi et al., 2015).

When examining the genetic background of the oxytocin receptor gene, dog breeds differ from wolves (*Canis lupus*) in the frequency of oxytocin receptor (OXTR) gene variations (Bence et al., 2016), microsatellite markers close to the OXTR gene (Oliva et al., 2016), and OXTR methylation patterns (Banlaki et al., 2017). Methylation levels at certain sites in the OXTR promoter region were found to be different in Border collie females than in males. In males, methylation levels associate with a higher likelihood to approach a threatening unfamiliar person, and a lower likelihood to remain passive or hide behind the owner (Cimarelli et al., 2017).

On a genetically varied sample, Ottenheimer-Carrier et al. (2017) did not find any relationship between two OXTR SNP-s and behavior/personality measures in dogs. The authors suggested that links between OXTR gene SNPs and behavior might be breed-specific. Indeed, on single-breed samples, Kis et al. (2014) reported that three SNPs in the 5′ and 3′ untranslated regions (*UTR*) of the OXTR gene were associated with social behaviors toward humans (namely proximity seeking and friendliness, which were composite traits based on behavioral tests). OXTR gene polymorphisms have also been analyzed in another study conducted on golden retrievers. However, polymorphisms were not linked with separation-related behaviors in this breed (van Rooy et al., 2016).

The oxytocin system interacts with the opioid system that modulates reward, motivation, emotional responses, cognition, nociception, and autonomic functions. In females, opioids inhibit oxytocin release, especially at mu and kappa receptors (Vuong et al., 2010), depending on reproductive state (Evans and Olley, 1988). Opioid-oxytocin interactions probably have anatomical basis (Keverne, 2005). Higham et al. (2011) found that lactating rhesus macaque females possessing the G allele of the mu opioid receptor gene C77G SNP had higher cerebrospinal fluid oxytocin levels (but not different maternal behavior), than homozygous C females.

Several other studies implicate a role for endogenous opioid peptides (e.g., endorphins) in forming stable social relationships, such as pair bonding and attachment [e.g., in prairie voles (Burkett et al., 2011), domestic fowls (Warnick et al., 2005), and zebra finches (Schnelker et al., 2015)].

Morphine was the first chemical discovered to bind to mu opioid receptors. Genetic variations in the mu 1 opioid receptor genes (OPRM1) may primarily explain individual variation in the development of social relations in humans [parent-child interaction (Copeland et al., 2011); rejection by social partners (Way et al., 2009)].

In captive rhesus macaques, functional polymorphisms in the OPRM1 gene have been identified that are associated with social behaviors. Infants carrying the G allele of the C77G SNP exhibited higher levels of attachment behavior and higher distress to separation from their mothers, and they spent more time with their mothers upon reunion than individuals homozygous for the C allele. C/G infants were also less likely to interact with other individuals in the group (Barr et al., 2008). Female rhesus macaques with the G allele held their infants more (Higham et al., 2011).

In dogs, low doses of exogenous opiates have been found to significantly reduce distress vocalization and activity in socially isolated puppies (Panksepp et al., 1978). In a recent study, 34 SNP markers within the 500 kb region around the dog homolog of the OPRM1 gene were analyzed in golden retrievers affected by separation anxiety, and in control dogs, but no significant associations were observed (van Rooy et al., 2016). Two SNPs (C15A and C207T) in the exon 1 of the mu opioid receptor gene have been also studied. According to a preliminary study, dogs carrying the C allele of the C15A SNP have a greater susceptibility for dysphoric state following anesthesia. The authors also concluded that dog breeds closely related to wolves might be predisposed to dysphoria (Hawley and Wetmore, 2010).

In this study, we present data on genetic associations between the oxytocin and mu opioid receptor gene variants and greeting behavior in dogs. Dog breeds and breeding lines differ in several aspects of their social behavior toward humans (for a review see Mehrkam and Wynne, 2014). For example, while herding dog breeds were selected for cooperative work with continuous visual contact with their human partners, Siberian huskies, and other sled dogs were selected for work with no human visual contact (Gácsi et al., 2009). With regards to behavior and gene polymorphism associations, one could expect two different patterns of results. First, it could be hypothesized that all polymorphisms of a gene have the same effect in each breed/breeding lines, and thus in dogs in general. Second, it is also possible that the association patterns differ between populations. Specifically, some polymorphisms may have an effect in one population but not in another, or they have opposite effects in different breeds (due to gene-gene and gene-environment interactions).

Previous findings support the latter, more complex scenario. Hejjas et al. (2007) reported that a variable number of tandem repeats (VNTR) polymorphism in exon 3 of the dopamine D4 receptor gene (DRD4), was associated with activity-impulsivity in police German shepherd dogs (GSD), but not in pet GSDs. The authors suggested that the more homogenous environment in the case of police dogs helped the emergence of the subtle effects of the DRD4 polymorphism. However, it is also possible that the two populations had different ancestries (working line vs. show line); therefore, their genetic background was slightly different. On the other hand, the lack of association with one DRD4 polymorphism does not not necessarily mean that DRD4 is not involved in the behavior of pet GSDs. In a subsequent study, Hejjas et al. (2009) found that intron 2 VNTR of the same gene (DRD4) was associated with greeting behavior (referred to as “social impulsivity”) in pet GSDs (police dogs were not studied in this case). Interestingly, both behaviors has been reported to be linked with DRD4 in racing Siberian huskies (Wan et al., 2013). Although the allele frequency of DRD4 exon 3 is different between GSDs and Siberian huskies, an association with activity behavior score (including activity level during greeting a human) and a marginally significant association with the activity-impulsivity scale has been found in this breed. In sum, we can conclude that despite the genetic isolation of each breed/population and/or the allelic heterogeneity between breeds, the link between DRD4 and activity-impulsivity related measures seems to be generalizable to some breeds of dogs. Similar finding has been reported with regard to the tyrosine-hydroxylase gene (TH) and activity-impulsivity association which has been found to be significant in in pet GSDs (Kubinyi et al., 2012), and marginally significant in racing Siberian huskies (Wan et al., 2013).

The above findings suggest that with some divergence, gene-behavior associations might be similar between breeds. However, the study of Kis et al. (2014) reveals a more complicated picture. The authors found that one OXTR SNP had the same effect in the two herding breeds investigated, but another OXTR SNP had opposite effects in the breeds. Specifically, while carrying the G allele of the −213A/G SNP (formerly^1^: –212A/G) was associated with lower proximity seeking in both German shepherd dogs and Border collies, in the case of the 19208A/G SNP (formerly^2^: 19131AG) the lack of G allele predicted higher friendliness scores in German shepherd dogs, and the lack of A allele was linked to higher friendliness score in Border collies. (Because of linkage disequilibrium, the result was similar in the case of the rs8679684 SNP).

Certainly, the allele frequencies that are typical for a breed could modify the association patterns. Nevertheless, the question remains: how generalizable are gene-behavior associations in dogs?

In this study, we used the Greeting Test behavioral data collected by Kis et al. (2014) on herding dog breeds (Border collie and German shepherd dog) and Wan et al. (2013) on a non-herding dog breed (Siberian husky). The Greeting Test followed the same protocol in the two studies, and both were the first test presented in the test batteries. However, the scoring of the two tests were slightly different, so the raw data of Kis et al. (2014) was modified to be in harmony with the Wan et al. (2013) data (see Methods). We performed association studies between the assessed genetic variants (OXTR −213A/G, 19208A/G, rs8679684, and OPRM1 rs21912990) and the greeting behavior of the dogs. Finding similar or different patterns of gene-behavior associations in herding and non-herding dogs could imply that some OXTR SNPs are linked to social behavior in dogs in general, while others are breed-specific.

We also ran two pilot studies, which are presented in the Supplementary Material. The first is the genotyping of the kappa opioid receptor gene (OPRK1) rs23478162 SNP, and the description of the allele frequencies in three dog breeds and in wolves. The second is a comparative analysis on the expression levels of several opioid receptor genes (OPRM1, OPRD1, and OPRK1) conducted in various brain areas of a male beagle dog (see Supplementary Material).

## Materials and methods

### Subjects

One hundred and three border collies (1–12-years-old; mean age ± *SD*: 4 ± 3 years; male: 45%) and 104 German shepherd dogs (1–11-years-old; mean age ± *SD*: 4 ± 2 years; male: 57%) were assessed in a Greeting Test by Kis et al. (2014), and 96 Siberian huskies participating in sled dog races (1–14-years-old; mean age ± *SD*: 5 ± 3 years; male: 56%) by Wan et al. (2013).

None of the subjects were closely related, i.e., littermate and parent-offspring relationships were excluded.

For assessing the allele frequencies of the opioid receptor genes, 42 gray wolves (male: 45%) living in Hungarian, Serbian, Austrian, and German zoos and parks were also genotyped but did not participate in the Greeting Test.

### Buccal sample collection and DNA isolation and genotyping

Buccal samples were collected from subjects in a non-invasive way, with cotton swabs from the inner surface of the cheek. Genomic DNA was isolated as described in a previous study (Kotyuk et al., 2013).

We investigated the three OXTR SNPs studied by Kis et al. (2014). The genotyping of −213A/G, 19208A/G (formerly −212A/G and 19131A/G, respectively) and rs8679684 was as described in Kis et al. (2014).

Mu opioid receptor gene (OPRM1) rs21912990 SNP (NCBI, NC_006583.3) was genotyped by allele specific amplification using 5′ATG CAT CTC TAC TAC TAA GG 3′forward and 5′ TTT ACC TCC CTT CTC TTA TC 3′ reverse primers. For genotyping the rs21912990 SNP C allele specific: 5′GGC AGC CCT TCC ATG ATC 3′ and T allele specific: 5′GGC AGC CCT TCC ATG ATT 3′ primers were used. Annealing temperature was 52°C. The PCR products were analyzed by 1.5% agarose gel electrophoresis.

The genotyping of kappa opioid receptor gene (OPRK1) rs23478162 SNP is described in the Supplementary Material.

### Behavioral data

#### Greeting test

**Figure 1:**
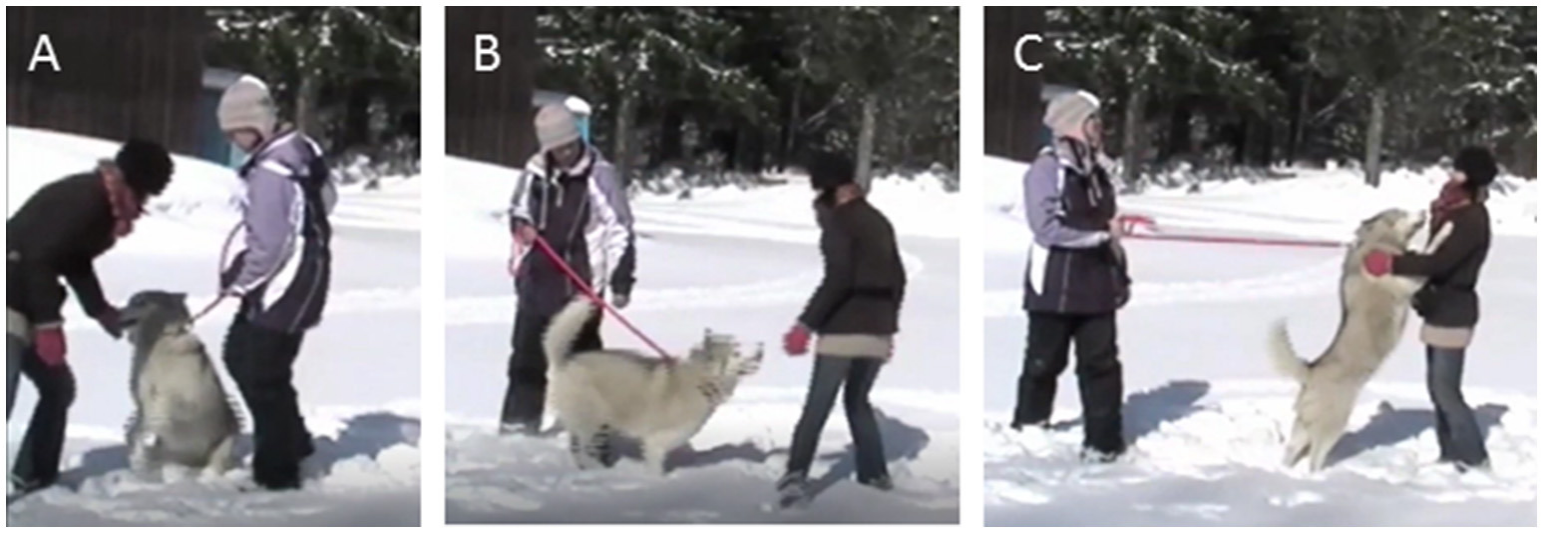
Sequence of video frames from a Greeting Test. **(A)** Experimenter approaches the non-aggressive dog. **(B)** Experimenter steps sideways, **(C)** Experimenter waits 2–3 s to check whether the dog followed her.

A female, unfamiliar experimenter greeted the dog, who was kept on leash by the owner (Figure 1).

In an open, undisturbed area, the owner stood in place while holding the dog on a 1.5–2 m leash. The dog was allowed to move freely within the range of the stretched leash and was not corrected or rewarded for any behavior. A female experimenter (unfamiliar to the dog) approached the dog in a friendly manner (verbally greeted the owner and the dog and smiled). When the dog acted “friendly” (moved toward the experimenter with affiliative behaviors) or showed neutral behavior, the experimenter stepped toward the dog and patted its head, back, and shoulders. Then she stepped 1 m sideways within reach of the leash and waited 2–3 s in order to check whether the dog followed her. If the dog showed aggressive behavior (e.g., barking or growling), the experimenter stayed out of reach of the leash, crouched, and tried to call the dog. If the dog approached the experimenter and was not aggressive, she followed the protocol above. If it was not possible to approach the dog, the test was terminated in 30 s.

In the study of Kis et al. (2014) the Greeting Test was coded with two behavioral variables: “Latency of approaching” and “Latency of following' the experimenter, on a 0–3 scale: 0: 0 s; 1: 1–5 s; 2: 5–15 s; 3: does not approach. For the present analysis, these scores were re-calculated, by applying the coding method of the “Approaches” variable in Wan et al. (2013). This simple coding system enabled Wan et al. (2013) to assess the behavior of numerous dogs in a short time, at the experimental site, which was especially important in the case of the racing Siberian huskies, which were tested around the racing area. Specifically: the “greeting score” in the present study was calculated as follows: we gave 0 points if both “Latency of approaching” and Latency of following' were 3 in the Kis et al. (2014) study (i.e., the dog did not approach the experimenter and could not be patted); 1 point if one of the variables was 0–2 in the Kis et al. (2014) study (i.e., the dog approached the experimenter once in a non-aggressive way); 2 points if both variables were 0–2 in the Kis et al. (2014) study (i.e., the dog approached the experimenter in a non-aggressive way both during their initial encounter and followed her when she stepped away).

Previous studies have shown that the “Latency of approaching” and “Latency of following” variables of the Greeting Test correlated with approaching the experimenter in other contexts (separation from the owner, calling after threatening, Kis et al., 2014). The “Approaches” variable loaded highly on the sociability factor together with contact with the experimenter, body posture and tail wagging variables of the Greeting Test (Wan et al., 2013). Interobserver reliabilities have been reported in the studies of Kis et al. (2014) and Wan et al. (2013).

### Statistical analysis

SNPs deviating from Hardy-Weinberg equilibrium or with minor allele frequency below 0.05 were removed. SPSS 21.0 for Windows was used for all statistical analyses. General Linear Models (GLM) were used to test the effects of age (as covariate), sex, and SNP (with SNK *post-hoc* test) as main effects on behavior (greeting score) with backward elimination on variable selection. We adjusted p values for multiple comparisons with Bonferroni's adjustment: 0.017 for border collies and GSDs (3 GLM-s were run), 0.025 for Siberian huskies (2 GLMs).

## Results

### OXTR variation and behavior

The descriptive statistics of the greeting score are shown in Table 1.

**Table 1:** Total number of participants in the Greeting Test, greeting score frequencies (%), and means for the three dog populations.

	**Total *N***	**Score 0 (*N*)**	**Score 1 (*N*)**	**Score 2 (*N*)**	**Mean score (*SD*)**
Border collie	103	5.8 (6)	16.5 (17)	77.7 (80)	1.72 (0.57)
German shepherd dog	104	2.9 (3)	11.5 (12)	85.6 (89)	1.83 (0.45)
Siberian husky	96	8.3 (8)	26.0 (25)	65.6 (63)	1.57 (0.64)

Table 2 presents the OXTR SNP genotype frequencies, allele frequencies, and Hardy-Weinberg equilibrium for each breed. In the Siberian husky breed the A allelic variant of the OXTR rs8679684 SNP was infrequent, and the −213A/G genotype frequencies deviated from Hardy-Weinberg equilibrium (chi2 = 9.94, *p* = 0.002), therefore these SNP-s were omitted from further analysis. In Border collies and GSDs rs8679684 and 19208A/G are in strong linkage disequilibrium (Kis et al., 2014), therefore the analyses were run only with 19208A/G.

**Table 2:** OXTR SNP genotypes (GT), number of individuals by genotype (*N*), genotype frequencies (%), allele frequencies, chi2 scores, and chi2 test *p*-values in three dog populations.

	**–213A/G**			**rs8679684**			**19208A/G**		
	**GT**	**N**	**%**	**GT**	**N**	**%**	**GT**	**N**	**%**
**Border collie**	AA	6	7	AA	1	1	AA	1	1
	AG	28	31	AT	7	7	AG	8	8
	GG	57	63	TT	88	92	GG	94	91
Allele freq	G	0.78		T	0.95		G	0.95	
**chi2**		0.96			3.25			2.61	
***p***		0.33			0.07			0.11	
**German shepherd dog**	AA	12	12	AA	40	40	AA	38	38
	AG	48	49	AT	46	46	AG	49	49
	GG	37	38	TT	15	15	GG	14	14
Allele freq	G	0.63		T	0.38		G	0.38	
**chi2**		0.35			0.09			0.08	
***p***		0.55			0.77			0.78	
**Siberian husky**	AA	17	22	AA	0	0	AA	0	0
	AG	23	30	AT	1	1	AG	12	15
	GG	37	48	TT	74	99	GG	68	85
Allele freq	G	0.63		T	0.99		G	0.93	
**chi2**		9.94			0.00			0.53	
***p***		0.00			0.95			0.47	

**Figure 2:**
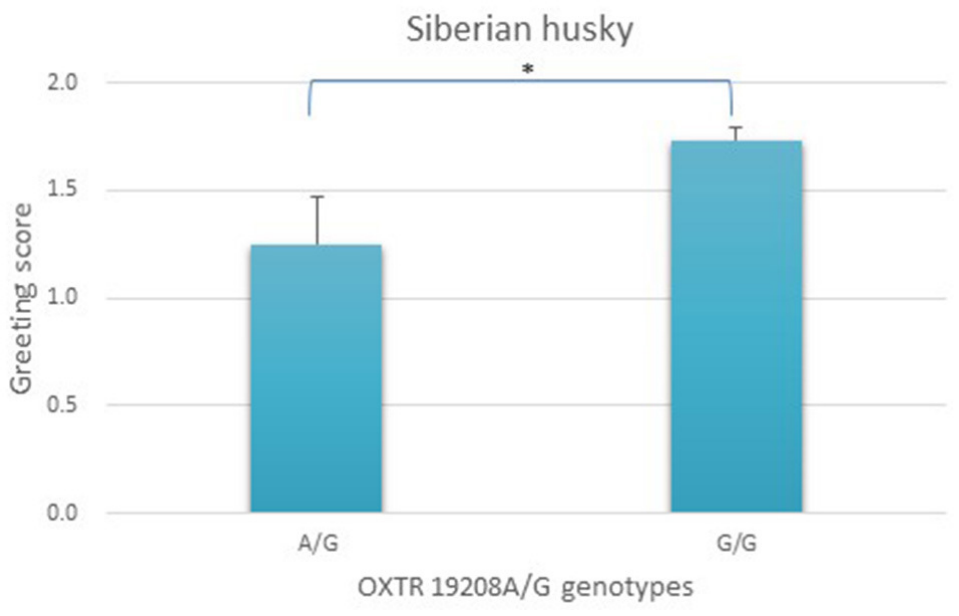
Association between OXTR 19208A/G genotypes and greeting scores (mean + SE) in Siberian huskies (*N* = 80). Genotype categories only included the G/G and the A/G groups, as the A/A genotype was missing the sample. The greeting behavior scores of G/G homozygotes was significantly higher than the score of dogs possessing the minor “A” allele (A/G). ^*^p < 0.05.

We did not find an age or sex effect in any of the populations. One gene-behavior association was detected in Siberian huskies, and the results of the association analysis are presented in Figure 2. Genotype categories only included the G/G and the A/G groups, as the A/A genotype was missing in our relatively small sample. According to the results, the greeting behavior scores of G/G homozygotes for the OXTR 19208A/G SNP was significantly higher than the score of dogs possessing the rare allele (A/G) [*F*_(1, 78)_ = 6.786, original *p* = 0.011, adjusted *p* = 0.022, partial eta squared = 0.08, Figure 2], demonstrating the behavioral effect of the rare genetic variant.

### OPRM1 variation and behavior

The OPRM1 rs21912990 SNP was not in linkage disequilibrium with the OXTR SNP-s in either breed (*p* = 0.34–0.81). The genotype frequencies of Siberian huskies and wolves were similar and C/C genotypes were more frequent than in Border collies and German shepherd dogs (chi2 = 84.533, *p* < 0.001).

The genotype distribution, allele frequencies and Hardy-Weinberg equilibriums are presented in Table 3. The measured genotypes of Border collies were not in Hardy-Weinberg equilibrium, but because p value was higher than 0.01 (*p* = 0.03) we performed the association analysis.

**Table 3:** OPRM rs21912990 genotype (GT), number of individuals by genotype (*N*), genotype frequencies (%), allele frequencies, chi2 scores, and chi2 test *p*-values in three dog populations and wolves.

	**OPRM1**		
	**GT**	***N***	**%**
**Border collie**	CC	27	33
	CT	47	58
	TT	7	9
Allele freq	T	0.38	
**chi2**		4.51	
***p***		0.03	
**German shepherd dog**	CC	11	20
	CT	27	50
	TT	16	30
Allele freq	T	0.55	
**chi2**		0.00	
***p***		0.95	
**Siberian husky**	CC	68	78
	CT	18	21
	TT	1	1
Allele freq	T	0.11	
**chi2**		0.02	
***p***		0.87	
**Wolf**	CC	31	74
	CT	11	26
	TT	0	0
Allele freq	T	0.13	
**chi2**		0.95	
***p***		0.33	

Association analysis of behavioral and genetic data on the OPRM1 rs21912990 SNP was conducted in all three dog breeds. There was no association in Siberian huskies [*F*_(1, 85)_ = 1.665, original *p* = 0.200, partial eta squared = 0.019].

**Figure 3:**
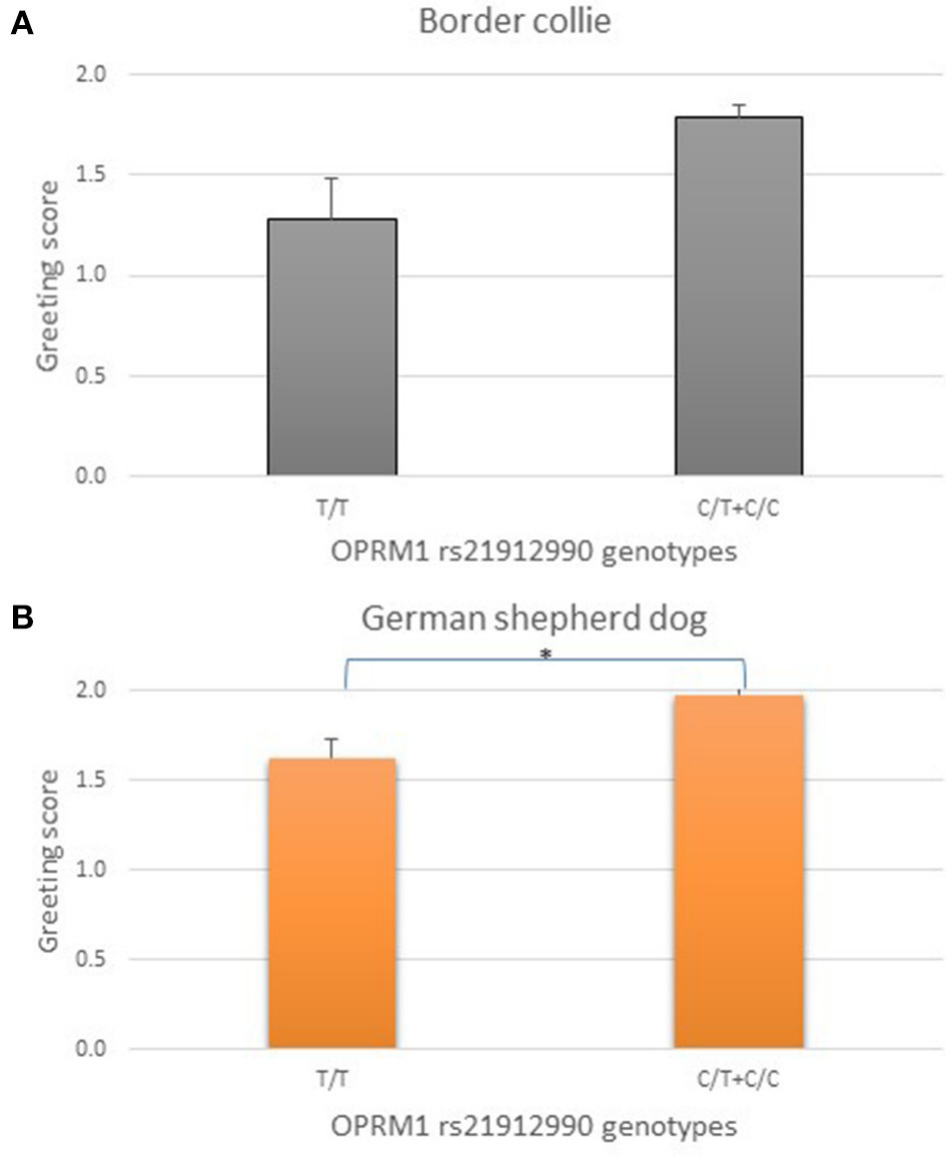
Association between OPRM1 rs21912990 genotypes and greeting scores (mean + SE) in Border collies (**A**, *N* = 81) and in German shepherd dogs (**B**, *N* = 54). T/T homozygotes tended to have lower scores than C/T and/or C/C dogs, therefore, the dominant model was applied comparing the genotype groups with the presence or absence of the dominant C allele. T/T homozygotes had marginally lower scores in Border collies, and significantly lower scores in German shepherd dogs than C/T and C/C individuals. ^*^p < 0.05.

Results of the genetic association analysis for the two herding breeds and OPRM1 rs21912990 SNP are shown in Figure 3. The genotypes were grouped according to the presence (C/C or C/T) or absence (T/T) of the major allele (C), as the behavioral data of major allele carriers were very similar. Border collies and German shepherd dogs without the major C allele (T/T) had lower greeting scores.

In Border collies OPRM1 rs21912990 T/T homozygotes tended to have lower scores than C/T and C/C dogs [*F*_(2, 78)_ = 2.819, original *p* = 0.066, partial eta squared = 0.067]. C/T and C/C genotypes did not differ. Therefore, the dominant model was applied comparing the genotype groups with the presence (C/C and C/T) or absence (T/T) of the dominant allele. This resulted in a trend toward significance after adjusting p for multiple comparisons [*F*_(1, 79)_ = 5.705, original *p* = 0.019, adjusted *p* = 0.057, partial eta squared = 0.067, Figure 3A].

In German shepherd dogs, OPRM1 rs21912990 T/T homozygotes had lower scores than C/T heterozygotes [*F*_(2, 51)_ = 4.221, original *p* = 0.020, partial eta squared = 0.142]. In accordance with the finding among Border collies, the dominant feature of the C allele was also shown among German shepherd dogs. Moreover, applying the dominant model for statistical analysis improved the significance of genetic differences [*F*_(1, 52)_ = 8.160, original *p* = 0.006, adjusted *p* = 0.018, partial eta squared = 0.136, Figure 3B].

## Discussion

In this study, we assessed the potential association of oxytocin and opioid receptor gene SNPs with greeting an unfamiliar human in three dog populations: pet Border collies, pet German shepherd dogs, and racing Siberian huskies. The genes were selected as candidates because they have been implicated in the development of complex social behaviors in mammals (e.g., Copeland et al., 2011).

We found that Siberian huskies that lacked the C allele of the OXTR 19208A/G SNP had higher scores in the Greeting test, i.e., they approached a friendly unfamiliar woman more frequently in a non-aggressive way. The same polymorphism was linked to the friendliness behavioral scale in the study of Kis et al. (2014). However, the friendliness scale did not include variables from the greeting test, as it was composed of the variables describing reactions to a threatening and a passive stranger. According to the results of the study by Kis et al. (2014) and the present study, the lack of A allele is associated with increased friendliness in Border collies, and more frequent approach during greeting in Siberian huskies, while the lack of G allele of the 19208A/G is linked to increased friendliness in German shepherd dogs.

In the Kis et al. (2014) study, the variables measured in the Greeting test were part of the proximity seeking scale, which was found to be associated with the –213A/G SNP in both herding breeds. However, this SNP was not in Hardy-Weinberg equilibrium in the Siberian husky population, therefore it was omitted from the investigation.

In conclusion, although the OXTR gene seems to be associated with the broad dimension of human directed social behavior, the precise variables of this dimension may or may not be related to the specific polymorphisms of the gene in different breeds. Thus, the relationship between behavior and genes is complex and breed specific. This is not surprising if we consider that (1) behavior traits are usually associated with many genetic variants, each of which has only a subtle effect on the behavior, thus gene-gene and gene-environment interactions could easily obscure them; (2) the relationship between genetic variations and allele frequencies are different between isolated dog populations with different ancestries; (3) alleles may not directly affect the phenotype, but are correlated with a causative allele located nearby—and the relationship between locations differ between breeds.

In the present study, we investigated not only OXTR, but another gene, which has been implicated in social behavior, the OPRM1. The allele frequencies of the rs21912990 SNP were similar in Siberian huskies and gray wolves, but differed from that of Border collies and German shepherd dogs, which might reflect their genetic relatedness and/or more similar social behavior.

The OPRM1 rs21912990 SNP tended to associate with greeting behavior in Border collies and was also significantly linked to this behavior in German shepherd dogs. T/T homozygotes approached the unfamiliar human less frequently than other dogs. This result is in harmony with findings in rhesus macaques (Barr et al., 2008; Higham et al., 2011), which suggest that individuals with certain OPRM1 allelic variants differ in behavior relating to social affiliation. However, the association was missing in the Siberian husky breed, probably because there was only one individual with the T/T genotype. Once again, the pattern of results suggest that the link between gene polymorphisms and behavior are probably breed specific. Therefore, when considering the applied aspect of opioid receptor studies, it would be important to examine in several breeds how variations in responses to opioid administration may be influenced by these SNPs (Hawley and Wetmore, 2010).

Although the results should be regarded as preliminary due to the relatively low sample size, our results highlight how the social behavior of dogs toward humans is influenced by the oxytocin and opioid system, but the links between SNP-s and behavioral variables might differ by breeding populations.

## Ethics statement

Non-invasive studies on dogs are currently allowed to be done without any special permission in Hungary by the University Institutional Animal Care and Use Committee (UIACUC, Eötvös Loránd University, Hungary). The currently operating Hungarian law “*1998. évi XXVIII. Törvény*”—the Animal Protection Act—defines experiments on animals in the 9th point of its 3rd paragraph (3. §/9.). According to the corresponding definition by law, our non-invasive observational study is not considered to be an animal experiment. The owners volunteered to participate and approved of the genetic analyses and behavioral testing of their dogs.

## Author contributions

The idea for the paper was conceived by EK and ÁM. The experiments were designed by EK, MB, ZR, and MS. The experiments were performed by EK, MW, MB, DK, and EP. The data were analyzed by EK. The paper was written by EK, MB, and MS.

### Conflict of interest statement

The authors declare that the research was conducted in the absence of any commercial or financial relationships that could be construed as a potential conflict of interest.

## Footnotes

^1^Position number is determined according to the most recent genome version, this explains the change of nomenclature compared to Kis et al. (2014).

^2^See above.
