# Supplementary material for: Oxytocin and Opioid Receptor Gene Polymorphisms Associated with Greeting Behavior in Dogs
Source: Front Psychol. 2017 Sep 7;8:1520. doi: 10.3389/fpsyg.2017.01520 (PMC5594098; doi:10.3389/fpsyg.2017.01520)
Supplement: Supplementary file 1 [file Table1.pdf]

Frontiers in Psychology
Supplementary Material

Oxytocin and opioid receptor gene polymorphisms associated with greeting behavior in dogs

Enikő Kubinyi, Melinda Bence, Dora Koller, Michele Wan, Eniko Pergel, Zsolt Ronai, Maria Sasvari-Szekely, Ádám Miklósi

In the present study we report the kappa opioid receptor gene allele frequencies for three dog populations (pet Border collies, pet German shepherd dogs and racing Siberian huskies) and wolves (*Canis lupus*). Allele C was infrequent in all populations.

Subjects

Samples were collected from 103 pet Border collies, 104 pet German shepherd dogs, 96 racing Siberian huskies, and 42 captive grey wolves. For more details, see main text. In addition, we collected samples from 24 Labrador retrievers, 16 golden retrievers and 15 beagles (all pets).

Genotyping

Kappa opioid receptor gene (OPRK1) rs23478162 SNP (NCBI, NC_006611.3) was genotyped by PCR-RFLP method using 5'CAT GAG GGA TGT TGA TGG GCT 3' forward and 5'GGA AAC CAC CCT TTG CAG TTT 3' reverse primers. Annealing temperature was 52 °C. PCR products were incubated for 3 h at 37 °C in a restriction enzyme mixture containing 0.5 U/μl AvaI restriction enzyme (NEB), 1x BSA and 1x NEB4 buffer. The digested PCR products were analyzed by 1.5% agarose gel electrophoresis.

Results

OPRK1 variation

The genotype distribution, allele frequencies and Hardy-Weinberg equilibriums are presented in Table S1. Allele C was rare in all populations except in Labrador retrievers.

	OPRK1		
	**GT**	**N**	**%**
**Beagle**	CC	0	0
	CT	0	0
	TT	15	100
allele freq	T	**1.00**	
**chi2**		-	
**p**		-	
**Border collie**	CC	1	2
	CT	1	2
	TT	41	95
allele freq	T	**0.97**	
**chi2**		18.43	
**p**		0.00	
**German shepherd dog**	CC	0	0
	CT	0	0
	TT	56	100
allele freq	T	**1.00**	
**chi2**		-	
**p**		-	
**Golden retriever**	CC	0	0
	CT	0	0
	TT	100	100
allele freq	T	1	
**chi2**		-	
**p**		-	
**Labrador retriever**	CC	5	21
	CT	13	54
	TT	6	25
allele freq	T	**0.52**	
**chi2**		**0.17**	
**p**		0.68	
**Siberian husky**	CC	0	0
	CT	1	4.8
	TT	20	95.2
allele freq	T	0.98	
**chi2**		**0.01**	
**p**		0.01	
**Wolf**	CC	0	0
	CT	0	0
	TT	39	100
allele freq	T	**1.00**	
**chi2**		-	
**p**		-	

Table S1. OPRM rs21912990 genotype (GT), number of individuals by genotype (N), genotype frequencies (%), allele frequencies, chi2 scores and chi2 test p-values in three dog populations and wolves. Allele C was infrequent in all populations except in Labrador retrievers.
