# Supplementary material for: Oxytocin and Opioid Receptor Gene Polymorphisms Associated with Greeting Behavior in Dogs
Source: Front Psychol. 2017 Sep 7;8:1520. doi: 10.3389/fpsyg.2017.01520 (PMC5594098; doi:10.3389/fpsyg.2017.01520)
Supplement: Supplementary file 2 [file Image1.pdf]

Frontiers in Psychology
Supplementary Material

Oxytocin and opioid receptor gene polymorphisms associated with greeting behavior in dogs

Enikő Kubinyi, Melinda Bence, Dora Koller, Michele Wan, Eniko Pergel, Zsolt Ronai, Maria Sasvari-Szekely, Ádám Miklósi

We performed quantitative real-time PCR to assess expressional differences of the three major types of opioid receptors, μ (mu), δ (delta) and κ (kappa) (OPRM1, OPRD1, and OPRK1) genes in the prefrontal cortex, hippocampus, and amygdala of one male beagle. A 2 to 5-fold increase of OPRM1 expression in the hippocampus and amygdala have been found. We report the method to encourage more research on these receptors which are involved in emotional reactions and social behavior.

Introduction

The three major types of opioid receptors are distributed throughout the central nervous system and periphery. Each type belongs to a subfamily of G protein-coupled receptors (Lutz and Kieffer, 2013).
Mu and delta opioid receptors functionally interact *in vivo*. The two receptors are co-expressed in neurons from brain networks related to water and food consumption, sexual behavior, perception and responses to aversive stimuli (Erbs et al., 2014) .
The distribution and/or function of κ receptor may differ between sexes (Chartoff and Mavrikaki, 2015). κ receptor is involved in stress, depression, anxiety, dysphoria (Land et al., 2008), and substance dependence in humans (Gerra et al., 2007), as well as voluntary alcohol-drinking in mice (Vadasz et al., 2000).

Subject

For the pilot study brain samples from one male beagle dog euthanized on the owner's request were obtained at the Department of Anatomy and Histology, Faculty of Veterinary Science, Szent István University, Budapest, Hungary.

Reverse transcriptase (RT)-PCR analysis and real-time PCR

Total RNA samples from three brain regions (prefrontal cortex, amygdala and hippocampus) of one male beagle dog were isolated by RNeasy kit (Qiagen, Valencia, CA). The isolated RNA was treated with DNaseI enzyme (Thermo Fisher Scientific, Waltham, MA, USA). 1 µg RNA was reverse-transcribed with M-MLV reverse transcriptase (Thermo Fisher Scientific, Waltham, MA, USA) using random primers in 20 µL volume. Negative control was prepared without reverse transcriptase.
To determine mRNA expression of canine opiate receptors in different brain regions, quantitative real-time PCR assay were performed in 15 µL final volume containing 1 µL cDNA, 1× SYBR Green qPCR master mix (Thermo Fisher Scientific, Waltham, MA, USA), and 1-1 uM primers.
For amplification of the dog OPRM1 mRNA (NCBI, XM_003432544.1) 5' TCG TGT GCG TGG TGG GTC TC 3' forward and 5'TCT GGA AGG GCA GGG TAC TGG T 3' reverse primers were used.
For dog delta opioid receptor (OPRD1) mRNA (XM_544455.3) 5' TGG TCA TGG CTG TGA CCC GC 3' forward and 5' GAT GGG CAC GAC GAA GGC GA 3' reverse primers were used.

For OPRK1 mRNA (NCBI, XM_544080.3) 5' CGC ACA CCC ATG AAG GCA AAG A 3' forward and 5'TGG GAA TTG CAA GGA GCA CTC GAT 3' reverse primers were used. The primers were designed to span different exons resulting in 100-150 bp PCR products.

Expression levels of the dog HPRT mRNA were detected with TaqMan assay in 25 µL final volume containing 0.2 µL cDNA, 1× ABI PCR master mix, gene-specific TaqMan primers and FAM-labeled probe (Thermo Fisher Scientific, Waltham, MA, USA). Amplification and signal detection were performed using an ABI 7300 Real-Time PCR System (Thermo Fisher Scientific, Waltham, MA, USA). Denaturation at 95°C for 10 min was followed by 40 thermocycles (65°C, 15 sec and 60°C, 1 min). Reactions were performed in triplicate using RNase-free water as negative control. Expression levels of opiate receptors were first normalized to the HPRT internal control gene and then to expression levels measured in the prefrontal cortex. Results were expressed as fold changes calculated with the formula 2-ΔΔCT as in Bence et al. (2016).

Results and Discussion

Relative expression of OPRM1 gene in different brain areas

PCR product quality check by agarose gel electrophoresis showed no aspecific band or genomic DNA contamination. In accordance with previous results (Peckys and Landwehrmeyer, 1999), the expression patterns varied at the different brain regions (Figure S1). According to our raph estimates, the highest expression of OPRM1 was found in the amygdala, and a lower expression was found in the hippocampus, while the mRNA level in the canine cortex was very low. These preliminary results might support the role of this receptor in emotional reactions and social behavior (Adolphs, 2003).

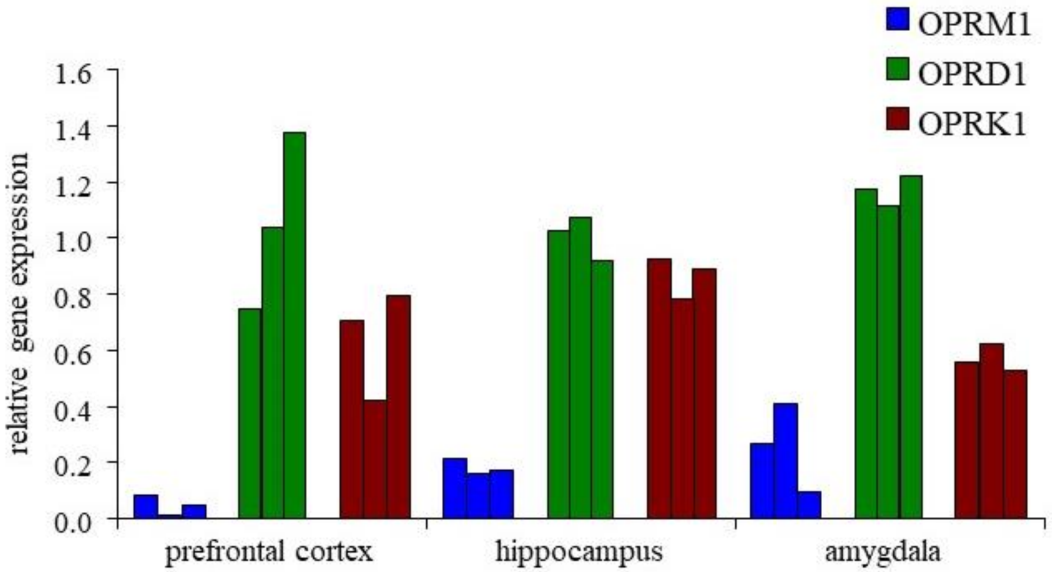

Figure S1. Relative gene expression levels of the different opiate receptor subtypes in various brain regions. Gene expression was measured by real-time quantitative PCR, and data were analyzed using the ΔC_T-method. Expression level of the HGPRT gene was investigated as an internal control for relative quantification. All measurements were carried out in triplicates. The figure shows the relative gene expression value of each individual measurement.

References

Adolphs, R. (2003). Cognitive Neuroscience of Human Social Behaviour. *Nat. Rev.* 4, 165–178. doi:10.1038/nrn1056.

Bence, M., Marx, P., Szantai, E., Kubinyi, E., Ronai, Z., and Banlaki, Z. (2016). Lessons from the canine Oxtr gene: populations, variants and functional aspects. *Genes, Brain Behav.*, 1–12. doi:10.1111/GBB.12356.

Chartoff, E. H., and Mavrikaki, M. (2015). Sex Differences in Kappa Opioid Receptor Function and Their Potential Impact on Addiction. *Front. Neurosci.* 9, 466. doi:10.3389/fnins.2015.00466.

Erbs, E., Faget, L., Scherrer, G., Matifas, A., Filliol, D., Vonesch, J. L., et al. (2014). A mu-delta opioid receptor brain atlas reveals neuronal co-occurrence in subcortical networks. *Brain Struct. Funct.*, 1–26. doi:10.1007/s00429-014-0717-9.

Gerra, G., Leonardi, C., Cortese, E., D'Amore, A., Lucchini, A., Strepparola, G., et al. (2007). Human Kappa opioid receptor gene (OPRK1) polymorphism is associated with opiate addiction. *Am. J. Med. Genet. Part B Neuropsychiatr. Genet.* 144B, 771–775. doi:10.1002/ajmg.b.30510.

Land, B. B., Bruchas, M. R., Lemos, J. C., Xu, M., Melief, E. J., and Chavkin, C. (2008). The dysphoric component of stress is encoded by activation of the dynorphin kappa-opioid system. *J. Neurosci.* 28, 407–14. doi:10.1523/JNEUROSCI.4458-07.2008.

Lutz, P.-E., and Kieffer, B. L. (2013). Opioid receptors: distinct roles in mood disorders. *Trends Neurosci.* 36, 195–206. doi:10.1016/j.tins.2012.11.002.

Peckys, D., and Landwehrmeyer, G. B. (1999). Expression of mu, kappa, and delta opioid receptor messenger RNA in the human CNS: a 33P in situ hybridization study. *Neuroscience* 88, 1093–1135. doi:10.1016/S0306-4522(98)00251-6.

Vadasz, C., Saito, M., Gyetvai, B., Mikics, E., and Vadasz, C. (2000). Scanning of five chromosomes for alcohol consumption loci. *Alcohol* 22, 25–34. doi:10.1016/S0741-8329(00)00098-7.
